# Reconstruction and analysis of the gene regulatory network for cell wall function in Arabidopsis thaliana L. leaves in response to water deficit

**DOI:** 10.18699/VJGB-23-118

**Published:** 2023-12

**Authors:** A.R. Volyanskaya, E.A. Antropova, U.S. Zubairova, P.S. Demenkov, A.S. Venzel, Y.L. Orlov, A.A. Makarova, T.V. Ivanisenko, T.A. Gorshkova, A.R. Aglyamova, N.A. Kolchanov, M. Chen, V.A. Ivanisenko

**Affiliations:** Institute of Cytology and Genetics of the Siberian Branch of the Russian Academy of Science, Novosibirsk, Russia Novosibirsk State University, Novosibirsk, Russia; Institute of Cytology and Genetics of the Siberian Branch of the Russian Academy of Science, Novosibirsk, Russia; Institute of Cytology and Genetics of the Siberian Branch of the Russian Academy of Science, Novosibirsk, Russia Novosibirsk State University, Novosibirsk, Russia; Institute of Cytology and Genetics of the Siberian Branch of the Russian Academy of Science, Novosibirsk, Russia Kurchatov Genomic Center of ICG SB RAS, Novosibirsk, Russia; Institute of Cytology and Genetics of the Siberian Branch of the Russian Academy of Science, Novosibirsk, Russia Kurchatov Genomic Center of ICG SB RAS, Novosibirsk, Russia; Institute of Cytology and Genetics of the Siberian Branch of the Russian Academy of Science, Novosibirsk, Russia I.M. Sechenov First Moscow State Medical University of the Ministry of Health of the Russian Federation (Sechenov University), Moscow, Russia Peoples’ Friendship University of Russia, Moscow, Russia; Institute of Cytology and Genetics of the Siberian Branch of the Russian Academy of Science, Novosibirsk, Russia; Institute of Cytology and Genetics of the Siberian Branch of the Russian Academy of Science, Novosibirsk, Russia Kurchatov Genomic Center of ICG SB RAS, Novosibirsk, Russia; Kazan Institute of Biochemistry and Biophysics, FRC Kazan Scientific Center of RAS, Kazan, Russia; Kazan Institute of Biochemistry and Biophysics, FRC Kazan Scientific Center of RAS, Kazan, Russia; Institute of Cytology and Genetics of the Siberian Branch of the Russian Academy of Science, Novosibirsk, Russia; College of Life Sciences, Zhejiang University, Hangzhou, China; Institute of Cytology and Genetics of the Siberian Branch of the Russian Academy of Science, Novosibirsk, Russia Novosibirsk State University, Novosibirsk, Russia Kurchatov Genomic Center of ICG SB RAS, Novosibirsk, Russia

**Keywords:** plant cell wall, drought, plants, differentially expressed genes, text mining, microarray, gene regulatory network, клеточная стенка растений, засуха, растения, дифференциально экспрессирующиеся гены, интеллектуальный анализ текста, микрочип, регуляторная генная сеть

## Abstract

The plant cell wall represents the outer compartment of the plant cell, which provides a physical barrier and triggers signaling cascades under the influence of biotic and abiotic stressors. Drought is a factor that negatively affects both plant growth and development. Cell wall proteins (CWP) play an important role in the plant response to water deficit. The adaptation mechanisms of the cell wall to water loss are of interest for identifying important genetic factors determining plant drought resistance and provide valuable information on biomarkers for further selection aimed at increasing the yield of crop plants. Using ANDSystem, a gene network describing the regulation of CWPs under water restriction conditions was reconstructed. The analysis of the gene network and the transcriptome data analysis allowed prioritizing transcription factors (TF) based on their enrichment of differentially expressed genes regulated by them. As a result, scores were calculated, acting as indicators of the association of TFs with water deficit. On the basis of the score values, eight most significant TFs were selected. The highest priority was given to the TF GBF3. CWPs were prioritized according to the criterion of summing up the scores of transcription factors regulating these genes. Among the most prioritized CWPs were the AT5G03350 gene encoding a lectin-like protein, AT4G20860 encoding BBE-like 22 required for the oxidation of cellulose degradation products, and AT4G37800 encoding xyloglucan endotransglucosylase/ hydrolase 7. Overall, the implemented algorithm could be used for prediction of regulatory interactions between transcription factors and target genes encoding cell wall proteins in plants.

## Abstract (ru)

Растительная клеточная стенка представляет собой внешний компартмент растительной клетки, который во многом обеспечивает физический барьер и запуск сигнальных каскадов при действии био- и абиотических стрессоров. Засуха негативно влияет как на рост, так и развитие растений. Белки клеточной стенки (БКС) играют существенную роль в ответе растений на водный дефицит. Механизмы адаптации клеточной стенки к потере воды могут быть использованы для выявления важных генетических факторов, определяющих устойчивость растений к засухе, и предоставляют ценные данные о биомаркерах для дальнейшей селекции, направленной на повышение урожайности культурных растений. С помощью ANDSystem реконструирована генная сеть, позволяющая описывать регуляцию БКС в условиях ограничения полива. Анализ генной сети совместно с анализом транскриптомных данных позволил провести приоритизацию транскрипционных факторов (ТФ) по их обогащенности регулируемыми дифференциально экспрессирующимися генами. В результате были рассчитаны веса, являющиеся индикаторами ассоциации ТФ с водным дефицитом. По значениям весов отобраны восемь наиболее значимых ТФ. Наибольшим приоритетом обладал ТФ GBF3. Приоритизация БКС проведена по критерию суммирования весов транскрипционных факторов, регулирующих эти гены. К наиболее приоритетным БКС отнесены ген AT5G03350, кодирующий лектин-подобный белок; AT4G20860, кодирующий фермент берберинового моста BBE-like 22 (berberine bridge enzyme-like 22), необходимый для окисления продуктов распада целлюлозы, и AT4G37800, кодирующий ксилоглюкан эндотрансгликозилазу/гидролазу 7.

## Introduction

The plant cell wall is a complex structure composed of numerous biopolymers. The structure and composition of the cell wall change during plant development and are incredibly diverse not only between plant species but also between tissue types (Burton et al., 2010). Throughout their life cycle, plants are exposed to abiotic stresses such as drought, flooding, salinity, heavy metal pollution, nutrient deficiencies, and more. The plant cell wall provides a structural basis for supporting plant growth, serves as a source of various signals, and contributes to plant resistance to stressors.

Drought is a significant environmental problem, severely affecting plant growth, development, and yield. Plants subjected to water deficit exhibit morphological changes, in which proteins that are part of the cell wall play a critical role (Le Gall et al., 2015; Ezquer et al., 2020). However, the functions of these proteins, their regulation, and their interactions require further investigation.

Plant adaptation to drought has been demonstrated to be mediated by signaling pathways involving transcription factors (TFs) (Singh, Laxmi, 2015; Joshi et al., 2016). Therefore, studying the role of TFs as the primary regulators of water deficit-sensitive genes is particularly interesting. TFs regulate the expression of water deficit-sensitive genes in an abscisic acid (ABA)-dependent or ABA-independent manner (Yamaguchi- Shinozaki, Shinozaki, 2006). ABA-dependent positive regulators include the ABF/AREB (ABA-responsive element (ABRE)-binding proteins/ABRE-binding factors) family of the bZIP (basic leucine zipper) type, which recognizes ABAsensitive elements (ABRE) in the promoters of ABA-induced genes (Choi et al., 2000). The ABA-dependent regulatory pathway also includes several other families of transcription factors, such as AP2/ERF, MYB, NAC, and bHLH. In contrast, key ABA-independent regulators are members of the DREB family (Fujita et al., 2011).

Reconstructing gene networks based on the analysis of transcriptomic data obtained under water deficit conditions can contribute to understanding the molecular-genetic mechanisms underlying the formation and functioning of the plant cell wall in drought resistance. Currently, approaches based on the automatic analysis of scientific publication texts are actively used for gene network reconstruction. Previously we have developed the cognitive ANDSystem tool based on artificial intelligence methods, which performs automatic extraction of knowledge from scientific publications and factographic databases (Ivanisenko et al., 2015, 2019, 2020, 2022a). ANDSystem has been applied to a wide range of tasks, including the interpretation of metabolomic data in the analysis of blood plasma from COVID-19 patients (Ivanisenko et al., 2022b) and the prioritization of genes associated with human diseases (Saik et al., 2016, 2018a, b, 2019; Yankina et al., 2018; Antropova et al., 2022). The ANDSystem technology has also been used to solve problems in the field of plant biology. For example, with the help of ANDSystem, the SOLANUM TUBEROSUM knowledge base (Saik et al., 2017; Ivanisenko et al., 2018), which contains associative gene networks of plants, was developed. The application of ANDSystem allowed the identification of important genes involved in the response to abiotic stresses caused by drought, soil salinity, and elevated cadmium concentration (Demenkov et al., 2021).

To date, several studies have been carried out on the reconstruction of gene networks describing the response of Betula platyphylla and barley to drought (Javadi et al., 2021; Jia et al., 2022). Gene networks have also been constructed that describe the biosynthesis of the secondary cell wall of A. thaliana and the interactions of TFs that regulate cell wall biosynthesis in rice (Taylor-Teeples et al., 2015; Zhao et al., 2019). However, these gene networks have not been focused on the involvement of the cell wall in response mechanisms to water deficiency.

Using the ANDSystem software package (Ivanisenko et al., 2015, 2019, 2020, 2022a), we reconstructed a gene network based on the analysis of transcriptomic data for Arabidopsis thaliana leaves under water deficit conditions (Perera et al., 2008; Ding et al., 2009; Kühn et al., 2014; Fang et al., 2016; Noman et al., 2019). The reconstructed gene network and transcriptomic analysis prioritized TFs and genes encoding cell wall proteins (CWP) based on their involvement in the stress response during water deficit. The method of transcription factor prioritization contributed to isolating key regulatory proteins that are sensitive to the effects of water deficiency. The identification of key TFs made it possible to identify a list of target genes involved in the mechanisms of cell wall resistance to water deficit conditions. The final gene network containing priority genes included 8 TFs and 59 protein genes present in the cell wall according to the WallProtDB database (San Clemente, Jamet, 2015). According to the prioritization results, the GBF3 gene encoding the TF made the most significant contribution to the regulation of cell wall genes. Among the cell wall genes, the lectin-like protein was the most important. The results reveal potential molecular-genetic mechanisms of the plant cell wall response to water deficit.

## Materials and methods

Identification of Arabidopsis thaliana cell wall proteins. The WallProtDB plant cell wall proteomics database (http:// www.polebio.lrsv.ups-tlse.fr/WallProtDB) (San Clemente, Jamet, 2015) was used for finding the A. thaliana cell wall proteins. WallProtDB contains proteins identified using mass spectrometry technology in the cell wall proteome. According to the WallProtDB data, all cell wall proteins were divided into nine functional classes: 1) proteins acting on cell wall carbohydrates, 2) oxidoreductases, 3) proteases, 4) proteins with protein or polysaccharide interaction domains, 5) structural proteins, 6) lipid metabolism-related proteins, 7) proteins presumably involved in signal transduction, 8) various proteins, and 9) proteins with unknown function (Jamet et al., 2008). Enzymes synthesizing cell wall components and forming necessary substrates are not included in the list, as they are localized in other compartments and are, therefore, not represented in the WallProtDB database.

Processing of transcriptomic data. Data on the differential expression of A. thaliana genes under limited watering conditions were taken from the DNA microarray experiments database from the NCBI Gene Expression Omnibus (GEO) (https://www.ncbi.nlm.nih.gov/geo/) (Perera et al., 2008; Noman et al., 2019; Fang et al., 2016; Ding et al., 2009; Kühn et al., 2014). Bioinformatics analysis of transcriptomic data was performed in the R programming environment using Bioconductor packages (Gentleman et al., 2004). Reading of the CEL files containing probe identifiers and intensities was done using the readAffy() function from the affy package (Gautier et al., 2004). Data normalization, background noise correction, and gene expression level calculations were done using the affy package’s rma() function. Differential gene expression analysis was performed using the limma package (Ritchie et al., 2015).

To identify differentially expressed genes (DEGs) across multiple experiments, consistently activated or consistently suppressed under water deficit conditions, binomial distribution (p-value = 0.05) was applied using the binomtest() function implemented in the scipy.stats library

Reconstruction and analysis of the gene network. The gene network describing regulatory relationships of TFs with target genes associated with the cell wall response of A. thaliana leaves to water deficit was constructed using the ANDSystem software package (Ivanisenko et al., 2015, 2019, 2020, 2022a).

Prioritization of transcription factors and their target genes.The prioritization of transcription factors was carried out based on the score of the transcription factor (STF) values. The STF for a given transcription factor was equal to the number of DNA microarray experiments in which the list of cell wall genes regulated by this TF was enriched with DEGs. Enrichment was assessed using the hypergeometric distribution.

The prioritization of cell wall genes was carried out using the score of cell wall protein (SCWP), equal to the number of connections between the cell wall gene and TFs in the gene network

## Results

General analysis scheme

**Fig. 1.**
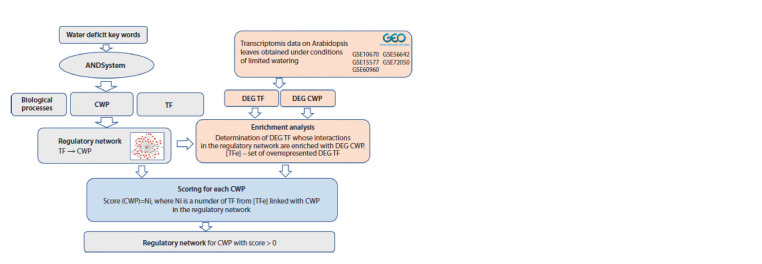
A pipeline for reconstructing a regulatory gene network describing the regulation of expression of A. thaliana cell wall proteins in drought response. DEG, differentially expressed genes; CWP, cell wall protein; TF, transcription factors.

The overall workflow is shown in Figure 1. It consists of the stage of transcriptome data analysis for A. thaliana leaves under water deficit conditions (this analysis aims to determine stably DEGs), gene network reconstruction stage (at this stage, the cell wall gene regulatory network under water deficit conditions was reconstructed using automated text analysis methods for scientific publications, factographic databases, and differential gene expression data), and the stage of prioritizing genes based on their involvement in the response to stress caused by water deficit.

Differentially expressed genes under water deficit conditions

To determine the DEGs of A. thaliana under limited watering conditions, an analysis of data from five DNA microarray experiments from the NCBI GEO (https://www.ncbi.nlm.nih. gov/geo/) was carried out (Table 1). All data were obtained on the Affymetrix Arabidopsis ATH1 Genome Array platform. In all experiments, the subject of the study was leaves, and the duration of days without watering ranged from 4 to 14 days.

**Table 1.**
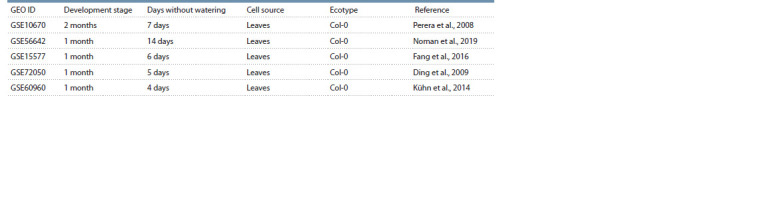
Publicly available DNA microarray data for A. thaliana leaves used in this study

A p-value threshold and a log fold change threshold (logFC) were used to determine differentially expressed genes: p- value < 0.05 and logFC > 1. Table 2 shows the number of DEGs for each of the five experiments. Expression data analysis across five experiments, performed using the binomial distribution, showed that changes in gene expression in two or more experiments could indicate that the gene is a stable DEG with a significance level of p-value < 0.05.

**Table 2.**
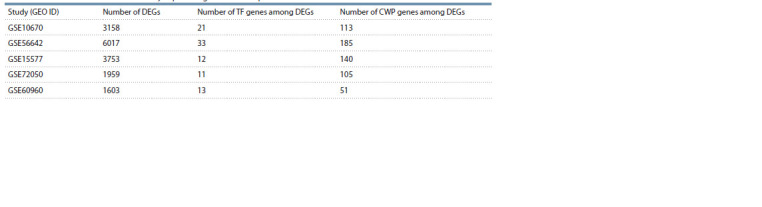
Number of identified differentially expressed genes in the experiments

Gene network reconstruction

The input data for the gene network reconstruction consisted of 1073 A. thaliana gene identifiers TAIR, encoding CWP, obtained from the WallProtDB database (Supplementary Table 11). Using ANDSystem, a regulatory network was reconstructed, containing interactions of these genes with transcription factors. For 692 genes, 599 potential TFs were identified. 381 CWP genes without interactions with TFs were removed from the network


Supplementary Materials are available in the online version of the paper: https://vavilovj-icg.ru/download/pict-2023-27/appx34.xlsx


**Fig. 2.**
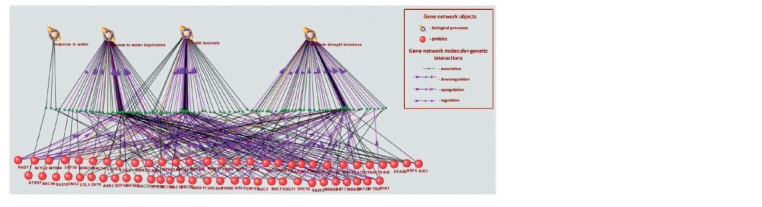
Associative network of transcription factors related to drought

In the next step, we selected TFs considering their involvement in biological processes related to plant responses to drought. In ANDSystem, four biological processes were represented, with their names containing the keywords “drought” and “water” in combination with “tolerance” and “deprivation.” These processes included the response to water deprivation, obsolete drought tolerance, drought recovery, and response to water. Fifty-six TFs were associated with these processes in ANDSystem (Fig. 2)

These 56 TFs were found to regulate 425 CWP genes (Suppl. Table 2). As a result of the analysis of A. thaliana leaves, it was shown that 23 TFs (Suppl. Figure) and 146 CWP genes demonstrated a stable unidirectional change in expression (Suppl. Table 3). However, not all CWP genes among the targets of the 23 TFs in the gene network were stably unidirectional DEGs. Therefore, we assessed the importance of TFs for the plant response to drought based on the enrichment analysis of their targets in the CWP DEG gene network. We assumed that the more CWP DEGs are among the targets of a transcription factor, the more significantly the transcription factor is associated with the plant’s response to water deficit.

Prioritization of transcription factors and their target genes and reconstruction of the resulting gene network

The prioritization of TFs and CWP genes for the response to drought was based on the STF and SCWP criteria, which characterized both their differential expression and their connections with DEGs in the gene network (see methods). The values of these indicators were calculated for the participants of the gene network and are presented in Supplementary Tables 4 and 5. The highest STF and SCWP values corresponded to the highest priority.

**Fig. 3.**
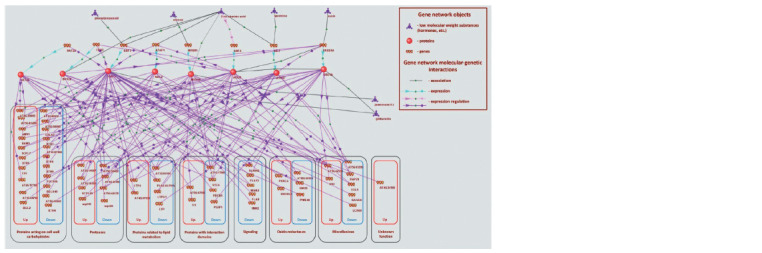
Gene network regulating the cell wall of Arabidopsis thaliana L. in response to water deficit and connection with key hormones.

Priority TFs were selected based on the statistically significant enrichment of their target genes encoding CWPs in DEGs in at least one of the transcriptomic experiments. Thus, according to this criterion, out of 23 TFs, 8 priority TFs were identified. The identified transcription factors belonged to the TF families HD-ZIP, bZIP, ERF, NAC, and MYB. All TFs were removed from the network to obtain the resulting gene network, except for those identified as priority TFs. Cell wall protein genes not connected to TFs were also removed. After filtering, the gene network contained 8 TFs and 59 CWP genes (see Suppl. Tables 4 and 5). We were also interested in analyzing the possible regulation of the identified TFs by active low-molecular-weight compounds (polyamines and hormones). For this purpose, the gene network was expanded with interactions of TFs with metabolites (Fig. 3).

## Discussion

The scientific literature actively studies the genetic regulation of plant cell wall functioning under drought conditions. To date, a large amount of information has been accumulated on the molecular-genetic events of plant responses to water deficit, including data from differential gene expression experiments. Applying an approach based on the reconstruction of gene networks allows for integrating disparate knowledge to describe the molecular-genetic mechanisms of complex cell processes. Here, we reconstructed a gene network using ANDSystem and prioritized its participants based on their importance for the response of A. thaliana to drought conditions. The reconstructed gene network contains eight transcription factor genes and their protein products, six low-molecularweight compounds (hormones and polyamines), and 59 genes encoding cell wall components regulated by the identified TFs (see Fig. 3). These 59 genes belong to 8 of the nine functional groups according to the classification presented in Supplementary Table 5. One functional class (structural protein group) is not represented among the identified genes.

The most prioritized transcription factor

Interestingly, all eight identified genes encoding TFs showed expression activation (see Suppl. Table 4). Among them, GBF3 regulates the transcription of the most significant number of genes encoding CWP. At the same time, it was stably expressed in all analyzed A. thaliana transcriptomes in this study obtained under water restriction conditions. There is also evidence that overexpression of GBF3 in A. thaliana led to improved resistance to osmotic stress, salinity, and drought, in addition to conferring insensitivity to ABA (Ramegowda et al., 2017).

Proteins acting on cell wall carbohydrates

In the reconstructed gene network, the largest group was represented by genes encoding proteins that act on cell wall carbohydrates (21 genes), 10 of which showed activation and 11 showed suppression of expression. This functional class includes expansins, glycosidases, and esterases. The highest priority among this functional class was given to the gene XTH7 (AT4G37800), encoding xyloglucan endotransglucosylase/ hydrolase 7. XTHs can hydrolyze and reconnect the molecules of xyloglucan – the key hemicellulose of primary cell walls (Rose et al., 2002). These enzymes are involved in cell wall remodeling during plant cell growth and response to various stressors. XTHs are encoded by a large multigene family, members of which are differentially expressed in various physiological situations having peculiarities in activity mode and regulation nuances (Zhang et al., 2017; Nazipova et al., 2022). According to the SCWP indicator, XTH7 is ranked third among the 59 considered cell wall genes (5 points, see Suppl. Table 5). As can be seen from Figure 3, this gene is regulated by TFs GTF3 and DREB1A (CBF3). The XTH7 enzyme and its activity have not been fully characterized yet. According to gene expression data, XTH7 is involved in salt resistance and ethylene-dependent apple softening (Zhang et al., 2017; Cai et al., 2023). It also participates in processes such as cell enlargement and restructuring. According to our differential expression analysis, it showed stable suppression, as a result of which its influence on limiting cell growth under drought conditions can be assumed. The other members of this functional class had priority ratings ranging from one to three points. The second-highest ranking gene is AT2G43570, encoding the enzyme endochitinase CHI (3 points, see Suppl. Table 5)

Proteases

The next most represented functional group was proteases, with eight genes. Subtilases are the most represented family of cell wall proteases (Jamet et al., 2008). Eight genes belonging to the protease functional group were identified in the regulatory gene network, with 4 showing activation of expression and 4 showing suppression. According to the SCWP indicator, three genes from this functional group scored three points: AT2G23000, encoding serine carboxypeptidaselike 10 (SCPL10), which is necessary for the biosynthesis of sinapoylated anthocyanins; AT3G14067, encoding subtilisinlike protease SBT1.4; and AT5G44530, encoding subtilisinlike protease SBT2.3 (see Suppl. Table 5). SBT1.4 is also called senescence-associated subtilisin protease due to its role in leaf aging. It has been shown that SCPL10 slows down the elongation of the main shoot, branching, and size of inflorescences (Martinez et al., 2015). According to our analysis, the expression of this gene increases under water deficit conditions, suggesting that SCPL10 may play a direct role in inhibiting plant growth under drought conditions. Other representatives of this group scored 1 to 2 SCWP points.

Proteins with interaction domains (with proteins or polysaccharides)

In our study, the highest SCWP score was obtained by the AT5G03350 gene (7 points, see Suppl. Table 5), which belongs to the functional class of proteins having interaction domains with proteins or polysaccharides. This class includes lectins and enzyme inhibitors, such as polygalacturonase inhibiting protein, pectin methylesterase, and protease inhibitors. Among the six differentially expressed genes of this functional group identified under water deficit conditions, the expression of four genes was suppressed, while two genes demonstrated activation of expression. In a previous study conducted on 220 microarray samples of A. thaliana available in the GEO, it was also shown that under drought conditions, the AT5G03350 gene, encoding salicylic acid-induced legume lectin-like pro-tein 1, was suppressed 7.9 times (Shaik et al., 2013). It seems to be involved in A. thaliana responses to multiple environmental stresses (including cold, high light, oxidative, ozone, and wound) and SA-mediated processes occurring in the effector-induced immune response (Armijo et al., 2013; Biswas et al., 2022). Due to the unusual structure of the legume lectin domain, proteins of this family may have a wide range of carbohydrate-binding specificity (Sharma et al., 1997), which possibly determines their diverse functions (including involvement in symbiosis, defense mechanisms against bacterial infection, enhanced tolerance against insects, salinity, and stomatal closure) (Van Holle et al., 2017). According to our results, the AT5G03350 gene is most significantly associated with TFs differentially expressed under water deficit conditions. These factors include HAT22, BH122, MYB44, ABF3, and ATHB7. Other representatives of this class scored between 1 and 3 SCWP points.

Oxidoreductases

In the reconstructed regulatory network, five oxidoreductase genes were identified; under water deficit conditions, the expression of two of these genes was activated, and three were suppressed. According to our study, the AT4G20860 gene ranks second in priority among the 59 investigated cell wall genes, with an SCWP of 5 (see Suppl. Table 5). AT4G20860 encodes berberine bridge enzyme-like 22 (BBE-like 22), which is necessary to oxidize cellodextrins (cellulose degradation products). Its role under limited irrigation conditions is unclear; however, it has been shown that A. thaliana with increased expression of the AT4G20860 gene product is more resistant to the Botrytis cinerea fungus, presumably because oxidized cellodextrins are a less valuable carbon source (Locci et al., 2019). In the reconstructed gene network, AT4G20860 may be regulated by TFs such as HAT22, GBF3, and ABF3 and is activated under water deficit conditions.

Other representatives of the oxidoreductase class – peroxidases – perform a dual function in plant cell walls: they contribute to the weakening of the cell wall by releasing hydroxyl radicals (OH-), which can cause polysaccharide scission (Schweikert et al., 2000) and increase wall rigidity by strengthening extensin cross-links and supporting lignification and suberization of the cell wall (Novaković et al., 2018).

Proteins related to lipid metabolism

In the reconstructed gene network, six genes encode proteins involved in the metabolism of cell wall lipids. According to the analysis we conducted, under water deficit conditions, the expression of two genes was enhanced, while the expression of four genes was suppressed. Various studies have shown that plants remodel lipid composition in response to drought (Gigon et al., 2004; Liu et al., 2021). In experiments on milk thistle, it was demonstrated that under drought conditions, PLA2-ALPHA (AT2G06925), which encodes a secretory phospholipase A2 enzyme, had reduced expression (Ghanbari Moheb Seraj et al., 2022), which is also evident in our results. Secreted PLA2s are low molecular weight calcium-dependent enzymes, which specifically hydrolyze the sn-2 position of phospholipids and can do that in an organized membrane (Mariani, Fidelio, 2019). They are involved in many cell wallrelated processes; for example, Arabidopsis PLA2-ALPHA is required for the trafficking of PIN-FORMED auxin efflux transporters to the plasma membrane (Lee et al., 2010). The PLA2-ALPHA gene scored 4 points on the SCWP indicator (4th place in Suppl. Table 5), meaning it is significantly associated with regulatory factors differentially expressed under water deficit conditions (HAT22 and GBF3).

Three other genes from this category, AT1G27950 for LTPG1 (SCWP 3), AT5G59310 for LTP4 (SCWP 2), and AT2G15050 for LTP7 (SCWP 1) (see Suppl. Table 5) encode lipid transfer proteins. AT1G27950 is a membrane-localized protein with a predicted GPI (glycosylphosphatidylinositol)- anchor domain. It extensively exports intracellular lipids (e. g., C29 alkane) to the surface to build the cuticular wax layer (Lee et al., 2009). AT5G59310 and AT2G15050 belong to non-specific lipid transfer proteins encoded by a large multigene family and occur only in land plants (Salminen et al., 2016). They are small proteins with a tunnel-like hydrophobic cavity that makes them suitable for binding and transport of phospholipids as well as galactolipids across membranes. LTPs are suggested to play a role in wax or cutin deposition in the cell walls (Salminen et al., 2016).

Signaling

Our study identified five genes encoding cell wall proteins involved in signal transduction. The expression of all considered genes was suppressed under water deficit conditions. Based on the SCWP indicator, among the genes of this functional group, the gene AT2G45470 (see Suppl. Table 5) scored the highest number of points (3), encoding fasciclin-like arabinogalactan protein 8 (FLA8). Numerous plant FLAs are chimeric proteins that contain moderately glycosylated arabinogalactan protein and one to two fasciclin domains with characteristic highly conserved sequence stretches of around 15 residues and a conserved central YH motif. FLAs are non-structural components of the cell wall, might be linked to cell wall polysaccharides, and interact with various cell surface receptors involved in various plant development processes, including cellulose biosynthesis (Seifert, 2018). FLA8 itself has been poorly characterized. The AT2G45470 gene is significantly associated with the transcription factor GBF3.

Another identified representative of the signal protein class is wall-associated kinase 2 (WAK2), encoded by the At1g21270 gene, scoring 2 points on the SCWP. Alongside WAK1, WAK2 is a cell wall receptor with an intracellular protein kinase domain, a transmembrane domain, and an extracellular N-terminal domain capable of binding polyand oligogalacturonans (Wagner, Kohorn, 2001). By binding pectins, WAK initiates signal transmission through mitogenactivated protein kinases (MAPK) for activation of vacuolar invertase and numerous other inducible proteins, regulating turgor pressure and, as a result, increasing cell size (Kohorn et al., 2006). Using antisense RNA, WAK2 is necessary for leaf cell expansion (but not for cell division) (Wagner, Kohorn, 2001). By interacting with polygalacturonan fragments formed as components of DAMP and PAMP under the influence of biotic and abiotic events, WAK can also trigger (via MAPK activation) a stress response. WAK expression is induced by injury, pathogen infection, and exposure to other stress factors such as ozone and heavy metals (Kohorn B.D., Kohorn S.L., 2012). A study conducted on sweet orange graft showed that WAK2 expression was suppressed under drought conditions in both sweet orange plants grafted on drought-tolerant and drought-sensitive rootstocks (Gonçalves et al., 2019). It can be assumed that under water deficit conditions, plants reduce WAK2 expression to lower turgor pressure, suspend leaf cell expansion, and induce other components of the stress response.

Miscellaneous

For this group, our study revealed seven genes – 5 with reduced and 2 with increased expression under water deficit conditions. Among them, based on the SCWP indicator, two genes scored 4 points each (5–6th places in Suppl. Table 5) – the downregulated AT5G15230, encoding the poorly characterized gibberellin-regulated protein 4 (GASA4), and the upregulated AT5G42510, encoding dirigent protein 1 (DIR1). DIR family proteins are involved in lignin and lignin biosynthesis and play a role in plant response to biotic and abiotic stresses (particularly drought) that cause physical damage to the cell wall (Paniagua et al., 2017). It has previously been shown that the expression of several genes encoding DIR proteins is sensitive to water and cold stress and treatment with ABA. Moreover, in Brassica plants under water stress, the increased expression of DIR genes was temporally coordinated with an increase in lignin content (Thamil Arasan et al., 2013). In Eucommia ulmoides Oliv seedlings, it was shown that the expression level of DIR1 increased almost 8-fold under osmotic stress within 6 hours and increased nearly three times under drought conditions within 12 hours (Li et al., 2021). The other representatives of this functional group each scored one point.

Hormones

Our reconstructed gene network also included hormones: ethylene, abscisic acid, auxin, jasmonate, gibberellin, and spermine – endogenous polyamine. These compounds affect transcription factors (altering the expression level or protein activity), subsequently leading to changes in the expression levels of target genes for transcription factors. Regulatory connections in the reconstructed network have been demonstrated under various conditions. Additional experiments are needed to explore whether they function under water deficit conditions.

For example, it was shown that upon infection of A. thaliana with aphids, the concentration of ethylene increases, which in turn induces the expression of the transcription factor MYB44 (Xia et al., 2014). Another transcription factor in our gene network, ABF3, is one of the key factors that transmit the abscisic acid signal and regulate the expression of target genes during water deficit (Yoshida et al., 2010). Under drought conditions, abscisic acid also induces the expression of the transcription factor ATHB7, which was observed within 30 minutes after experimentally induced stress, and ATHB7 transcription continued to increase after 21 hours (Söderman et al., 1996). The expression of the transcription factor GBF3 is also activated by abscisic acid (Lu et al., 1996).

Polyamine spermine is essential for plants to respond to drought, as demonstrated in mutant A. thaliana plants knocked out for genes encoding spermine-synthesizing enzymes (Yamaguchi et al., 2007). Under water deficit conditions, the stomata of such plants remained open. Another low-molecularweight compound in the gene network is gibberellin. Various studies have shown that reducing its level improves plant drought resistance (Shohat et al., 2021). Under cold conditions, the transcription factor DREB1A (CBF3) suppresses gibberellin accumulation (Zhou et al., 2017).

Based on the analysis of cell wall gene expression in different A. thaliana experiments under water deficit conditions, it can be noted that the plant’s response to this abiotic factor involves changes in the expression of genes encoding proteins from almost all functional groups characteristic of the cell wall. The exception was the group of structural proteins, which may indicate that changes in the composition of cell wall structural components in response to water deficit do not occur or occur to a negligible extent. It can be observed that the expression of 23 examined genes is enhanced under these conditions, while that of 36 is weakened. In each functional group, there are both activated and deactivated genes, except for the group of genes encoding signaling proteins, in which the expression of all five examined genes was suppressed under water deficit conditions.

## Conclusion

An analysis of five A. thaliana transcriptomes obtained under water deficit conditions was conducted. The implemented algorithm allowed to perform the prediction of potential regulatory interactions between transcription factors and target genes encoding cell wall proteins, which may play an important role in the response of A. thaliana to water deficit. Among the identified eight transcription factors regulating A. thaliana cell wall genes, GBF3 had the highest priority. Out of the 59 cell wall genes examined, the AT5G03350 gene, encoding a lectin-like protein, was identified as the most prioritized for association with differentially expressed transcription factors under water deficit conditions. It is associated with transcription factors such as HAT22, BH122, MYB44, ABF3, and ATHB7. Also highly significantly associated with transcription factors are the AT4G20860 gene, encoding BBE-like 22, which is necessary for the oxidation of cellulose degradation products (associated transcription factors – HAT22, GBF3, and ABF3), and AT4G37800, encoding xyloglucan endotransglucosylase/ hydrolase 7 (transcription factors GTF3 and DREB1A), among others. Overall, the proposed algorithm that has been used to analyze the gene network of cell wall proteins can be applied to other model plant species.

## Conflict of interest

The authors declare no conflict of interest.

## References

Antropova E.A., Khlebodarova T.M., Demenkov P.S., Venzel A.S.,
Ivanisenko N.V., Gavrilenko A.D., Ivanisenko T.V., Adamovskaya
A.V., Revva P.M., Lavrik I.N., Ivanisenko V.A. Computer
analysis of regulation of hepatocarcinoma marker genes hypermethylated
by HCV proteins. Vavilovskii Zhurnal Genetiki i Selektsii
= Vavilov Journal of Genetics and Breeding. 2022;26(8):733-
742. DOI 10.18699/VJGB-22-89 (in Russian)

Armijo G., Salinas P., Monteoliva M.I., Seguel A., García C., Villarroel-
Candia E., Song W., van der Krol A.R., Álvarez M.E., Holuigue L.
A salicylic acid-induced lectin-like protein plays a positive role in
the effector-triggered immunity response of Arabidopsis thaliana
to Pseudomonas syringae Avr-Rpm1. Mol. Plant Microbe Interact.
2013;26(12):1395-406. DOI 10.1094/MPMI-02-13-0044-R

Biswas S., Mondal R., Srivastava A., Trivedi M., Singh S.K., Mishra Y.
In silico characterization, molecular phylogeny, and expression
profiling of genes encoding legume lectin-like proteins under various
abiotic stresses in Arabidopsis thaliana. BMC Genomics. 2022;
23(1):480. DOI 10.1186/s12864-022-08708-0

Burton R.A., Gidley M.J., Fincher G.B. Heterogeneity in the chemistry,
structure and function of plant cell walls. Nat. Chem. Biol. 2010;
6(10):724-732. DOI 10.1038/nchembio.439

Cai H., Xu Y., Yan K., Zhang S., Yang G., Wu C., Zheng C., Huang J.
BREVIPEDICELLUS positively regulates salt-stress tolerance
in Arabidopsis thaliana. Int. J. Mol. Sci. 2023;24(2):1054. DOI
10.3390/ijms24021054

Choi H., Hong J., Ha J., Kang J., Kim S.Y. ABFs, a family of ABA-responsive
element binding factors. J. Biol. Chem. 2000;275(3):1723-
1730. DOI 10.1074/jbc.275.3.1723

Demenkov P.S., Oshchepkova Е.А., Demenkov P.S., Ivanisenko T.V.,
Ivanisenko V.A. Prioritization of biological processes based on the
reconstruction and analysis of associative gene networks describing the response of plants to adverse environmental factors. Vavilovskii
Zhurnal Genetiki i Selektsii = Vavilov Journal of Genetics and
Breeding. 2021;25(5):580-592. DOI 10.18699/VJ21.065 (in Russian)

Ding Y., Lapko H., Ndamukong I., Xia Y., Al-Abdallat A., Lalithambika
S., Sadder M., Saleh A., Fromm M., Riethoven J.J., Lu G.,
Avramov Z. The Arabidopsis chromatin modifier ATX1, the myotubularin-
like AtMTM, and the response to drought; a view from
the other end of the pathway. Plant Signal. Behav. 2009;4(11):1049-
1058. DOI 10.4161/psb.4.11.10103

Ezquer I., Salameh I., Colombo L., Kalaitzis P. Plant cell walls tackling
climate change: biotechnological strategies to improve crop adaptations
and photosynthesis in response to global warming. Plants.
2020;9(2):212. DOI 10.3390/plants9020212

Fang L., Su L., Sun X., Li X., Sun M., Karungo S.K., Fang S., Chu J.,
Li S., Xin H. Expression of Vitis amurensis NAC26 in Arabidopsis
enhances drought tolerance by modulating jasmonic acid synthesis.
J. Exp. Bot. 2016;67(9):2829-2845. DOI 10.1093/jxb/erw122

Fujita Y., Fujita M., Shinozaki K., Yamaguchi-Shinozaki K. ABA-mediated
transcriptional regulation in response to osmotic stress in
plants. J. Plant Res. 2011;124(4):509-525. DOI 10.1007/s10265-
011-0412-3

Gautier L., Cope L., Bolstad B.M., Irizarry R.A. affy–analysis of Affymetrix
GeneChip data at the probe level. Bioinformatics. 2004;
20(3):307-315. DOI 10.1093/bioinformatics/btg405

Gentleman R.C., Carey V.J., Bates D.M., Bolstad B., Dettling M., Dudoit
S., Ellis B., Gautier L., Ge Y., Gentry J., Hornik K., Hothorn T.,
Huber W., Iacus S., Irizzary R., Leisch F., Li C., Maechler M., Rossini
A.J., Sawitzki G., Smith C., Tierney L., Yang J., Zhang J. Bioconductor:
open software development for computational biology
and bioinformatics. Genome Biol. 2004;5(10):R80. DOI 10.1186/
gb-2004-5-10-r80

Ghanbari Moheb Seraj R., Tohidfar M., Azimzadeh Irani M., Esmaeilzadeh-
Salestani K., Moradian T., Ahmadikhah A., Behnamian M.
Metabolomics analysis of milk thistle lipids to identify droughttolerant
genes. Sci. Rep. 2022;12(1):12827. DOI 10.1038/s41598-
022-16887-9

Gigon A., Matos A.R., Laffray D., Zuily-Fodil Y., Pham-Thi A.T. Effect
of drought stress on lipid metabolism in the leaves of Arabidopsis
thaliana (ecotype Columbia). Ann. Bot. 2004;94(3):345-351. DOI
10.1093/aob/mch150

Gonçalves L.P., Boscariol Camargo R.L., Takita M.A., Machado M.A.,
Dos Soares Filho W.S., Costa M.G.C. Rootstock-induced molecular
responses associated with drought tolerance in sweet orange
as revealed by RNA-Seq. BMC Genomics. 2019;20(1):110. DOI
10.1186/s12864-019-5481-z

Ivanisenko V.A., Saik O.V., Ivanisenko N.V., Tiys E.S., Ivanisenko
T.V., Demenkov P.S., Kolchanov N.A. ANDSystem: an Associative
Network Discovery System for automated literature mining
in the field of biology. BMC Syst. Biol. 2015;9(Suppl. 2):S2. DOI
10.1186/1752-0509-9-S2-S2

Ivanisenko T.V., Saik O.V., Demenkov P.S., Khlestkin V.K., Khlestkina
E.K., Kolchanov N.A., Ivanisenko V.A. The SOLANUM
TUBEROSUM knowledge base: the section on molecular-genetic
regulation of metabolic pathways. Vavilovskii Zhurnal Genetiki i
Selektsii = Vavilov Journal of Genetics and Breeding. 2018;22(1):
8-17. DOI 10.18699/VJ18.325 (in Russian)

Ivanisenko V.A., Demenkov P.S., Ivanisenko T.V., Mishchenko E.L.,
Saik O.V. A new version of the ANDSystem tool for automatic extraction
of knowledge from scientific publications with expanded
functionality for reconstruction of associative gene networks by considering
tissue-specific gene expression. BMC Bioinformatics. 2019;
20(Suppl. 1):34. DOI 10.1186/s12859-018-2567-6

Ivanisenko T.V., Saik O.V., Demenkov P.S., Ivanisenko N.V., Savostianov
A.N., Ivanisenko V.A. ANDDigest: a new web-based module
of ANDSystem for the search of knowledge in the scientific literature.
BMC Bioinformatics. 2020;21(Suppl. 11):228. DOI 10.1186/
s12859-020-03557-8

Ivanisenko T.V., Demenkov P.S., Kolchanov N.A., Ivanisenko V.A.
The new version of the ANDDigest tool with improved ai-based
short names recognition. Int. J. Mol. Sci. 2022a;23(23):14934. DOI
10.3390/ijms232314934

Ivanisenko V.A., Gaisler E.V., Basov N.V., Rogachev A.D., Cheresiz
S.V., Ivanisenko T.V., Demenkov P.S., Mishchenko E.L., Khripko
O.P., Khripko Y.I., Voevoda S.M. Plasma metabolomics and gene
regulatory networks analysis reveal the role of nonstructural SARSCoV-
2 viral proteins in metabolic dysregulation in COVID-19
patients. Sci. Rep. 2022b;12(1):19977. DOI 10.1038/s41598-022-
24170-0

Jamet E., Albenne C., Boudart G., Irshad M., Canut H., Pont-Lezica R.
Recent advances in plant cell wall proteomics. Proteomics. 2008;
8(4):893-908. DOI 10.1002/pmic.200700938

Javadi S.M., Shobbar Z.-S., Ebrahimi A., Shahbazi M. New insights
on key genes involved in drought stress response of barley: gene
networks reconstruction, hub, and promoter analysis. J. Genet. Eng.
Biotechnol. 2021;19(1):2. DOI 10.1186/s43141-020-00104-z

Jia Y., Niu Y., Zhao H., Wang Z., Gao C., Wang C., Chen S., Wang Y.
Hierarchical transcription factor and regulatory network for drought
response in Betula platyphylla. Hortic. Res. 2022;9:uhac040. DOI
10.1093/hr/uhac040

Joshi R., Wani S.H., Singh B., Bohra A., Dar Z.A., Lone A.A., Pareek
A., Singla-Pareek S.L. Transcription factors and plants response
to drought stress: current understanding and future directions. Front.
Plant Sci. 2016;7:1029. DOI 10.3389/fpls.2016.01029

Kohorn B.D., Kobayashi M., Johansen S., Riese J., Huang L.F.,
Koch K., Fu S., Dotson A., Byers N. An Arabidopsis cell wall-associated
kinase required for invertase activity and cell growth. Plant J.
2006;46(2):307-316. DOI 10.1111/j.1365-313X.2006.02695.x

Kohorn B.D., Kohorn S.L. The cell wall-associated kinases, WAKs,
as pectin receptors. Front. Plant Sci. 2012;3:88. DOI 10.3389/fpls.
2012.00088

Kühn K., Yin G., Duncan O., Law S.R., Kubiszewski-Jakubiak S.,
Kaur P., Meyer E., Wang Y., Colas C., Giraud E., Narsai R., Whelan
J. Decreasing electron flux through the cytochrome and/or alternative
respiratory pathways triggers common and distinct cellular
responses dependent on growth conditions. Plant Physiol. 2014;
167(1):228-2250. DOI 10.1104/pp.114.249946

Le Gall H., Philippe F., Domon J.M., Gillet F., Pelloux J., Rayon C.
Cell wall metabolism in response to abiotic stress. Plants. 2015;4(1):
112-166. DOI 10.3390/plants4010112

Lee O.R., Kim S.J., Kim H.J., Hong J.K., Ryu S.B., Lee S.H., Ganguly
А., Сho H.-T. Phospholipase A2 is required for PIN-FORMED
protein trafficking to the plasma membrane in the Arabidopsis root.
Plant Cell. 2010;22(6):1812-1825. DOI 10.1105/tpc.110.074211

Lee S.B., Go Y.S., Bae H.J., Park J.H., Cho S.H., Cho H.J., Lee D.S.,
Park O.K., Hwang I., Suh M.C. Disruption of glycosylphosphatidylinositol-
anchored lipid transfer protein gene altered cuticular
lipid composition, increased plastoglobules, and enhanced susceptibility
to infection by the fungal pathogen Alternaria brassicicola.
Plant Physiol. 2009;150(1):42-54. DOI 10.1104/pp.109.137745

Li Z., Li B., Zhao Y., Zhao D. Cloning and characterization of the DIR1
promoter from Eucommia ulmoides Oliv and its response to hormonal
and abiotic stress. Plant Cell, Tissue Organ Cult. 2021;146:
313-322. DOI 10.1007/s11240-021-02070-x

Liu B., Wang X., Li K., Cai Z. Spatially resolved metabolomics and lipidomics
reveal salinity and drought-tolerant mechanisms of cottonseeds.
J. Agric. Food Chem. 2021;69(28):8028-8037. DOI 10.1021/
acs.jafc.1c01598

Locci F., Benedetti M., Pontiggia D., Citterico M., Caprari C., Mattei
B., Cervone F., De Lorenzo G. An Arabidopsis berberine bridge
enzyme-like protein specifically oxidizes cellulose oligomers and
plays a role in immunity. Plant J. 2019;98(3):540-554. DOI 10.1111/
tpj.14237

Lu G., Paul A.L., McCarty D.R., Ferl R.J. Transcription factor veracity:
is GBF3 responsible for ABA-regulated expression of Arabidopsis
Adh? Plant Cell. 1996;8(5):847-857. DOI 10.1105/tpc.8.5.847

Mariani M.E., Fidelio G.D. Secretory phospholipases A2 in plants.
Front. Plant Sci. 2019;10:861. DOI 10.3389/fpls.2019.00861

Martinez D.E., Borniego M.L., Battchikova N., Aro E.M., Tyystjärvi
E., Guiamét J.J. SASP, a Senescence-Associated Subtilisin Protease,
is involved in reproductive development and determination
of silique number in Arabidopsis. J. Exp. Bot. 2015;66(1):161-174.
DOI 10.1093/jxb/eru409

Nazipova A., Gorshkov O., Eneyskaya E., Petrova N., Kulminskaya A.,
Gorshkova T., Kozlova L. Forgotten actors: glycoside hydrolases
during elongation growth of maize primary root. Front Plant Sci.
2022;10(12):802424. DOI 10.3389/fpls.2021.802424

Noman M., Jameel A., Qiang W.D., Ahmad N., Liu W.C., Wang F.W.,
Li H.Y. Overexpression of GmCAMTA12 enhanced drought tolerance
in Arabidopsis and Soybean. Int. J. Mol. Sci. 2019;20(19):
4849. DOI 10.3390/ijms20194849

Novaković L., Guo T., Bacic A., Sampathkumar A., Johnson K. Hitting
the wall-sensing and signaling pathways involved in plant cell wall
remodeling in response to abiotic stress. Plants. 2018;7(4):89. DOI
10.3390/plants704008

Paniagua C., Bilkova A., Jackson P., Dabravolski S., Riber W., Didi V.,
Houser J., Gigli-Bisceglia N., Wimmerova M., Budínská E., Hamann
T., Hejatko J. Dirigent proteins in plants: modulating cell wall
metabolism during abiotic and biotic stress exposure. J. Exp. Bot.
2017;68(13):3287-3301. DOI 10.1093/jxb/erx141

Perera I.Y., Hung C.Y., Moore C.D., Stevenson-Paulik J., Boss W.F.
Transgenic Arabidopsis plants expressing the type 1 inositol 5-phosphatase
exhibit increased drought tolerance and altered abscisic
acid signaling. Plant Cell. 2008;20(10):2876-2893. DOI 10.1105/
tpc.108.061374

Ramegowda V., Gill U.S., Sivalingam P.N., Gupta A., Gupta C., Govind
G., Nataraja K.N., Pereira A., Udayakumar M., Mysore K.S.,
Senthil-Kumar M. GBF3 transcription factor imparts drought tolerance
in Arabidopsis thaliana. Sci. Rep. 2017;7(1):9148. DOI
10.1038/s41598-017-09542-1

Ritchie M.E., Phipson B., Wu D., Hu Y., Law C.W., Shi W., Smyth G.K.
limma powers differential expression analyses for RNA-sequencing
and microarray studies. Nucleic Acids Res. 2015;43(7):e47. DOI
10.1093/nar/gkv007

Rose J.K., Braam J., Fry S.C., Nishitani K. The XTH family of enzymes
involved in xyloglucan endotransglucosylation and endohydrolysis:
current perspectives and a new unifying nomenclature. Plant Cell
Physiol. 2002;43(12):1421-1435. DOI 10.1093/pcp/pcf171

Saik O.V., Ivanisenko T.V., Demenkov P.S., Ivanisenko V.A. Interactome
of the hepatitis C virus: Literature mining with ANDSystem.
Virus Res. 2016;218:40-48. DOI 10.1016/j.virusres.2015.12.003

Saik O.V., Demenkov P.S., Ivanisenko T.V., Kolchanov N.A., Ivanisenko
V.A. Development of methods for automatic extraction of
knowledge
from texts of scientific publications for the creation of
a knowledge base Solanum Tuberosum. Agricultural Biol. 2017;
52(1):63-74. DOI 10.15389/agrobiology.2017.1.63eng

Saik O.V., Demenkov P.S., Ivanisenko T.V., Bragina E.Y., Freidin M.B.,
Goncharova I.A., Dosenko V.E., Zolotareva O.I., Hofestaedt R.,
Lavrik I.N., Rogaev E.I. Novel candidate genes important for asthma
and hypertension comorbidity revealed from associative gene
networks. BMC Med. Genomics. 2018a;11(1):61-76. DOI 10.1186/
s12920-018-0331-4

Saik O.V., Demenkov P.S., Ivanisenko T.V., Bragina E.Y., Freidin M.B.,
Dosenko V.E., Zolotareva O.I., Choynzonov E.L., Hofestaedt R.,
Ivanisenko V.A. Search for new candidate genes involved in the comorbidity
of asthma and hypertension based on automatic analysis
of scientific literature. J. Integr. Bioinform. 2018b;15(4):20180054.
DOI 10.1515/jib-2018-0054

Saik O.V., Nimaev V.V., Usmonov D.B., Demenkov P.S., Ivanisenko
T.V., Lavrik I.N., Ivanisenko V.A. Prioritization of genes involved
in endothelial cell apoptosis by their implication in lymphedema
using an analysis of associative gene networks with ANDSystem.
BMC Med. Genomics. 2019;12(Suppl. 2):117-131. DOI 10.1186/
s12920-019-0492-9

Salminen T.A., Blomqvist K., Edqvist J. Lipid transfer proteins: classification,
nomenclature, structure, and function. Planta. 2016;
244(5):971-997. DOI 10.1007/s00425-016-2585-4

San Clemente H., Jamet E. WallProtDB, a database resource for plant
cell wall proteomics. Plant Methods. 2015;11(1):2. DOI 10.1186/
s13007-015-0045-y

Schweikert C., Liszkay A., Schopfer P. Scission of polysaccharides
by peroxidase-generated hydroxyl radicals. Phytochemistry. 2000;
53(5):565-570. DOI 10.1016/S0031-9422(99)00586-5

Seifert G.J. Fascinating fasciclins: A surprisingly widespread family
of proteins that mediate interactions between the cell exterior and
the cell surface. Int. J. Mol. Sci. 2018;19(6):1628. DOI 10.3390/ijms
19061628

Shaik R., Ramakrishna W. Genes and co-expression modules common
to drought and bacterial stress responses in Arabidopsis and rice.
PLoS One. 2013;8(10):e77261. DOI 10.1371/journal.pone.0077261

Sharma V., Surolia A. Analyses of carbohydrate recognition by legume
lectins: size of the combining site loops and their primary specificity.
J. Mol. Biol. 1997;267(2):433-445. DOI 10.1006/jmbi.1996.0863

Shohat H., Eliaz N.I., Weiss D. Gibberellin in tomato: metabolism, signaling
and role in drought responses. Mol. Horticulture. 2021;1(1):
15. DOI 10.1186/s43897-021-00019-4

Singh D., Laxmi A. Transcriptional regulation of drought response: a
tortuous network of transcriptional factors. Front. Plant Sci. 2015;
6:895. DOI 10.3389/fpls.2015.00895

Söderman E., Mattsson J., Engström P. The Arabidopsis homeobox
gene ATHB-7 is induced by water deficit and by abscisic acid. Plant
J. 1996;10(2):375-381. DOI 10.1046/j.1365-313X.1996.10020375.x

Taylor-Teeples M., Lin L., de Lucas M., Turco G., Toal T.W., Gaudinier
A., Young N.F., Trabucco G.M., Veling M.T., Lamothe R.,
Handakumbura P.P., Xiong G., Wang C., Corwin J., Tsoukalas A.,
Zhang L., Ware D., Pauly M., Kliebenstein D.J., Dehesh K., Tagkopoulos
I., Breton G., Pruneda-Paz J.L., Ahnert S.E., Kay S.A.,
Hazen S.P., Brady S.M. An Arabidopsis gene regulatory network
for secondary cell wall synthesis. Nature. 2015;517(7536):571-575.
DOI 10.1038/nature14099

Thamil Arasan S.K., Park J.I., Ahmed N.U., Jung H.J., Hur Y.,
Kang K.K., Lim Y.P., Nou I.S. Characterization and expression analysis
of dirigent family genes related to stresses in Brassica. Plant
Physiol. Biochem. 2013;67:144-153. DOI 10.1016/j.plaphy.2013.
02.030

Van Holle S., De Schutter K., Eggermont L., Tsaneva M., Dang L.,
Van Damme E.J.M. Comparative study of lectin domains in model
species: new insights into evolutionary dynamics. Int. J. Mol. Sci.
2017;18(6):1136. DOI 10.3390/ijms18061136

Wagner T.A., Kohorn B.D. Wall-associated kinases are expressed
throughout plant development and are required for cell expansion.
Plant Cell. 2001;13(2):303-318. DOI 10.1105/tpc.13.2.303

Xia X., Shao Y., Jiang J., Ren L., Chen F., Fang W., Guan Z., Chen S.
Gene expression profiles responses to aphid feeding in chrysanthemum
(Chrysanthemum morifolium). BMC Genomics. 2014;15(1):
1050. DOI 10.1186/1471-2164-15-1050

Yamaguchi K., Takahashi Y., Berberich T., Imai A., Takahashi T., Michael
A.J., Kusano T. A protective role for the polyamine spermine
against drought stress in Arabidopsis. Biochem. Biophys. Res. Commun.
2007;352(2):486-490. DOI 10.1016/j.bbrc.2006.11.041

Yamaguchi-Shinozaki K., Shinozaki K. Transcriptional regulatory networks
in cellular responses and tolerance to dehydration and cold
stresses. Annu. Rev. Plant Biol. 2006;57(1):781-803. DOI 10.1146/
annurev.arplant.57.032905.105444

Yankina M.A., Saik O.V., Ivanisenko V.A., Demenkov P.S., Khusnutdinova
E.K. Evaluation of prioritization methods of extrinsic apoptotic
signaling pathway genes for retrieval of the new candidates associated
with major depressive disorder. Rus. J. Genet. 2018;54:1366-
1374. DOI 10.1134/S1022795418110170

Yoshida T., Fujita Y., Sayama H., Kidokoro S., Maruyama K., Mizoi J.,
Shinozaki K., Yamaguchi-Shinozaki K. AREB1, AREB2, and ABF3
are master transcription factors that cooperatively regulate ABRE dependent ABA signaling involved in drought stress tolerance and
require ABA for full activation. Plant J. 2010;61(4):672-685. DOI
10.1111/j.1365-313X.2009.04092.x

Zhang Z., Wang N., Jiang S., Xu H., Wang Y., Wang C., Li M., Liu J.,
Qu C., Liu W., Wu S., Chen X., Chen X. Analysis of the xyloglucan
endotransglucosylase/hydrolase gene family during apple fruit ripening
and softening. J. Agric. Food Chem. 2017;65(2):429-434. DOI
10.1021/acs.jafc.6b04536

Zhao K., Lin F., Romero-Gamboa S.P., Saha P., Goh H.J., An G.,
Jung K.H., Hazen S.P., Bartley L.E. Rice genome-scale network
integration reveals transcriptional regulators of grass cell wall synthesis.
Front. Plant Sci. 2019;10:1275. DOI 10.3389/fpls.2019.
01275

Zhou M., Chen H., Wei D., Ma H., Lin J. Arabidopsis CBF3 and
DELLAs
positively regulate each other in response to low temperature.
Sci. Rep. 2017;7(1):39819. DOI 10.1038/srep39819

